# Immunotherapy – 2082. Long term prevention of asthma and rhinitis in children with atopic dermatitis four year after discontinuation of sublingual immunotherapy

**DOI:** 10.1186/1939-4551-6-S1-P163

**Published:** 2013-04-23

**Authors:** Giovanni Passalacqua, Valentina Garelli, Francesca Sclifò, G Walter Canonica, Giovanni Pajno

**Affiliations:** 1 Dept of Internal Medicine, Genoa University Allergy and Respiratory Diseases, Genova, Italy; 2 Dept of Pediatrics, Allergy Unit, University of Messina, Messina, Italy

## Background

Atopic Dermatitis is a disease with high prevalence in Italy (about 15% of the population). As known, Atopic Dermatitis is often associated with respiratory allergy to mite.We previously showed that sublingual immunotherapy (SLIT) to mite has a beneficial clinical effect in patients with allergic extrinsic Atopic Dermatititis (AD). To investigate the long term effects, we performed a 4-year follow up after the termination of SLIT.

## Methods

46 children with AD, aged 5-16 years and allergic to mite were re-assessed 4 years after discontinuation of a 2-year course of SLIT (or placebo). Methacholine provocation tests were carried out at baseline, after 2 years (when SLIT was stopped) and after 4 years. The development of asthma and rhinitis was assessed by clinical evaluations.

## Results

The children who previously had received SLIT had significantly less asthma after 6 years as evaluated by clinical symptoms: odd ratio 3.73 (0.130 vs 0.511; P=0.02) as compared to the former placebo group. In addition, significantly less patients reported an increase in asthma scores P= 0.003. A significant difference in bronchial methacholine responsiveness was found between the two groups (P=0.03), with an overall lower bronchial hyperreactivity in children who previously received active SLIT.

## Conclusions

A 2-year course of SLIT to mite provided a long term clinical effect and prevented the development of asthma in children with allergic extrinsic form of Atopic Dermatitis.

